# Prevalence and factors associated with frailty among incident kidney transplant patients: a cross-sectional study

**DOI:** 10.1590/1516-3180.2024.0141.R1.29012025

**Published:** 2025-05-26

**Authors:** Raoni de Oliveira Domingues-da-Silva, Helady Sanders-Pinheiro, Emiliana Holanda Pedrosa, Camila Mendes dos Santos, Jerônimo Junqueira, Ronaldo de Matos Esmeraldo, Paula Frassinetti Castelo Branco Camurça Fernandes, Claudia Maria Costa de Oliveira, Tainá Veras de Sandes-Freitas

**Affiliations:** I Faculty of Medicine, Universidade Federal do Ceará (UFC), Fortaleza (CE), Brazil.; II Department of Internal Medicine/Nephrology, Faculty of Medicine, Universidade Federal de Juiz de Fora (UFJF), Juiz de Fora (MG), Brazil.; III Professional Master’s Degree in Transplantation, Universidade Estadual do Ceará (UECE), Fortaleza (CE), Brazil.; IV Postgraduate Program in Medical Sciences, Universidade Estadual do Ceará (UECE), Fortaleza (CE), Brazil.; V Hospital Universitário Walter Cantídio (HUWC) and Hospital Geral de Fortaleza (HGF), Fortaleza (CE), Brazil.; VI Head of the Kidney Transplant Program, Hospital Geral de Fortaleza (HGF), Fortaleza (CE), Brazil.; VII Head of the Urinary System Unit, Hospital Universitário Walter Cantídio (HUWC), Fortaleza (CE), Brazil; Professor, Professional Master’s Degree in Transplantation, Universidade Estadual do Ceará (UECE), Fortaleza (CE), Brazil.; VIII Head of the Kidney Transplant Program, Hospital Universitário Walter Cantídio (HUWC), Fortaleza (CE), Brazil; Professor, Professional Master’s Degree in Transplantation, Universidade Estadual do Ceará (UECE), Fortaleza (CE), Brazil.; IX Department of Internal Medicine/Nephrology, Faculty of Medicine, Universidade Federal do Ceará (UFC), Fortaleza (CE), Brazil; Professor, Professional Master’s Degree in Transplantation, Universidade Estadual do Ceará (UECE), Fortaleza (CE), Brazil; Assistant physician, Hospital Geral de Fortaleza, Fortaleza (CE), Brazil.

**Keywords:** Kidney transplantation, Frailty, Cognitive dysfunction, Activities of daily living, Cross-sectional studies, Solid Organ Transplantation, Physical Disability, Fried Scale, Katz Scale, Lawton and Brody Scale, MoCA Test

## Abstract

**BACKGROUND:**

Evidence on frailty prevalence in Brazilian patients with kidney transplant (KT) is scarce.

**OBJECTIVES:**

To estimate frailty prevalence in pre-KT patients and its association with functional, cognitive, and laboratory anomalies.

**DESIGN AND SETTING:**

Cross-sectional descriptive study included adult KT candidates assessed within 24 hours before KT, at two medical centers in Northeast Brazil.

**METHODS:**

Frailty was classified as non-frail (scores 0–1), intermediate frail (score 2), or frail (scores 3–5), using Fried et al. criteria. Patients were divided into: Non-frail (0–1) and Frail (≥ 2) groups. Katz and Lawton’s scales assessed the dependence on basic (ADLs) and instrumental (IADLs) activities of daily living, respectively. Montreal Cognitive Assessment was used to evaluate cognition. Laboratory tests were performed during pre-KT evaluation.

**RESULTS:**

Among 82 patients, most were male (80.5%), mixed-race (76.8%), and 48.8 ± 14.9-years-old. The Frail group (63.4%) comprised 34.1% intermediate frail, and 29.3% frail individuals. This group had a higher prevalence of hypertension (90.4% vs. 70%, P = 0.018), rheumatological diseases (15.4% vs. 0%, P = 0.024), cognitive impairment (71.0% vs. 40.7%, P = 0.020), dependence on ADLs (32% vs. 0%, P < 0.001) and IADLs (82% vs. 56.7%, P = 0.014), lower hemoglobin (11.9 ± 2.7 g/dL vs. 13.4 ± 1.8 g/d, P = 0.005), and lower creatinine levels (7.1 mg/dL, IQR 6–10 vs. 9.1 mg/dL, IQR 7–11, P = 0.044).

**CONCLUSIONS:**

Pretransplant frailty was prevalent and associated with functional disability, cognitive impairment, and biomarkers indicating sarcopenia. Early frailty assessment and identification of modifiable factors are essential.

## INTRODUCTION

Frailty phenotype is defined as “a biologic syndrome of decreased reserve and resistance to stressors, resulting from a cumulative decline across multiple physiologic systems, causing vulnerability to adverse outcomes”.^1^ Originally delineated within the context of aging, younger adults with chronic kidney disease (CKD) face elevated frailty risk, increasing with the decline in glomerular filtration rate. Chronic inflammation, anemia, malnutrition, sarcopenia, and inactivity have emerged as probable factors implicated in its pathogenesis.^2^ The frailty phenotype has been reported as a predictor of worse outcomes in patients with CKD,^2,3^ and includes physical and cognitive decline. Among individuals with end-stage kidney disease (ESKD), frailty is associated with a lower chance of listing and a lower success rate of kidney transplants (KTs).^4^ Following a KT, frailty is linked to an increased incidence of delayed graft function,^5,6^ post-transplant delirium,^7^ prolonged hospital stays,^6^ early hospital readmission, and increased mortality.^6,8^

The prevalence of frailty in patients with CKD varies considerably depending on the chosen diagnostic instrument and the diverse social, demographic, and clinical characteristics of the analyzed population. To comprehensively understand this condition, it is imperative to study populations with diverse epidemiological conditions. Among patients with ESKD undergoing dialysis treatment, the prevalence of frailty ranges from 14 to 73% in international cohorts,^2^ with similar findings reported in Brazilian studies (16.1% to 73.8%).^9-11^ In patients listed for KT, who are considered the healthier among those on dialysis, evidence is limited, with a prevalence rate ranging from 11 to 20%.^12^ Brazil currently ranks fourth in the world for the absolute number of KTs,^13^ performing 5,000 to 6,000 procedures per year, thus providing an opportunity to explore frailty. To the best of our knowledge, only one Brazilian single-center study has assessed frailty among waitlisted patients, using the diagnostic tool outlined by Fried et al.,^1^ 16.1% were considered frail, and 20.7% were considered intermediately frail.^11^

Frailty presents substantial challenges for prevention and treatment owing to its multifactorial and intricate nature. Although a comprehensive understanding of all pathophysiological pathways is yet to be achieved, identifying risk factors and correlates has proven invaluable in clinical practice, aiding in risk stratification and treatment of frail patients.^12^ Lower physical performance and cognitive deficits are common among KT candidates and increase the risk of mortality post-transplantation.^12^ Despite their significance, these conditions are frequently excluded from pre-KT evaluations because of the requirements of trained staff and time. However, addressing these conditions can yield significant benefits, particularly in the case of physical performance, which is modifiable.^12^

## OBJECTIVE

This study aimed to assess the prevalence of the physical frailty phenotype and its association with functional disabilities, cognitive dysfunction, and laboratory findings among waitlisted individuals with ESKD considered fit for KT, at two Brazilian transplant centers.

## PATIENTS AND METHODS

### Study design and population

This cross-sectional descriptive study involved KT candidates evaluated within 24 hours before undergoing KT surgery. The study was conducted between March 2019 and January 2021 at two medical centers located in a capital city of Northeast Brazil. Both institutions are tertiary-level care, education, and research centers that perform over 100 KT procedures annually, most of them with deceased donors. The study included adult patients (aged > 18 years) eligible for living or deceased KT, selected based on local allocation criteria and deemed clinically suitable for kidney transplantation. The exclusion criteria included patients with paresis/plegia, severe tremors of the extremities, disorders affecting hand functionality, visual impairment, severe cognitive deficits, or any other condition hindering the motor execution of the tests required for frailty diagnosis. Patients whose KT was not performed for any reason were excluded.

### Instruments and definitions

Demographic and clinical variables— age (years, < and ≥ 65 years), sex, race (Caucasian, mixed, afro-Brazilian, Asian), educational level (illiterate, intermediate school, high school, college), time on dialysis (months), CKD etiology (hypertension, diabetes mellitus, glomerulopathy, polycystic kidney disease, unknown, others), comorbidities (chronic heart failure, hypertension, diabetes mellitus, depression, pneumopathy, rheumatic disease), polypharmacy (use of ≥ 5 drug classes), body mass index (kg/m^2^), and donor source (living, deceased)— were evaluated through interviews and medial record consultations.

Frailty phenotype was accessed using the instrument proposed by Fried et al.^1^ This instrument consists of a five-domain test: slowness (measured via walking speed), weakness (measured by grip strength), unintentional weight loss (loss of ≥ 4 kg/year), self-reported exhaustion, and low physical activity (measured with the Minnesota Leisure Time Activity^14^ questionnaire). Each positive test score was 1, and the comprehensive frailty score was derived by summing the individual component scores, which ranged from 0 to 5. Candidates scoring 0 or 1 were classified as non-frail, 2 as intermediate frail, and 3–5 as frail, as proposed by McAdams-Demarco et al. for patients with ESKD.^15^ The study population was categorized into two groups: those who were not frail (Non-frail group, scored 0–1) and those who were either intermediate or frail (Frail group, scored ≥ 2), as reported by others,^11^ since intermediately frail patients already exhibit a risk for adverse outcomes.^16,17^

Cognitive status was evaluated using the Montreal Cognitive Assessment (MoCA)^18^ test, and a diagnosis of cognitive impairment was assigned to patients scoring 23 or below.^19^ Patients were deemed to be dependent in basic daily activities if they scored ≥ 1 on the Katz^20^ scale. Dependence in instrumental daily activities was identified by a score < 21 on the Lawton and Brody^21^ scale.

Laboratory tests routinely conducted as part of the pretransplant evaluation were also assessed. Complete blood count and creatinine, calcium, phosphorus, and C-reactive protein levels were obtained as part of routine admission immediately before the KT. Parathyroid hormone, vitamin D, lipid, and iron profiles were collected during the semi-annual outpatient routine and were considered as the most recent measurements.

### Statistical analysis

Descriptive statistics of the whole sample and the two groups (Frail and Non-frail) was performed. Categorical variables are presented as frequencies and percentages; groups were compared using Chi-square or Fischer tests. The Shapiro–Wilk test was used to assess continuous data distribution patterns. Normally distributed continuous variables are summarized as means and standard deviations; groups were compared using the Student’s t-test. Non-normally distributed continuous variables were described as medians and interquartile ranges (IQRs) and compared using the Mann–Whitney U test. R software (version 4.2.2, R Core Team, Vienna, Austria, 2023) was used for statistical analyses, and differences were considered significant at P value < 0.05.

### Ethical considerations

The study was reviewed and approved by the Institutional Review Boards (IRB) of the Hospital Universitário Walter Cantídio and Hospital Geral de Fortaleza (approval numbers 3.485.900 and 3.158.457, on August 5, 2019 and February 21, 2019, respectively). Informed consent was obtained from all patients before the procedures were initiated. Patient records and information were anonymized and de-identified prior to analysis.

## RESULTS

### Population and Demographics

During the study period, 369 KT procedures were performed at the two transplantation centers. Among these, 22 were conducted on pediatric patients, 52 declined participation in the study, 34 were excluded because of challenges in completing some frailty diagnostic tests, and 179 were excluded because the research team was unable to perform the study tests before the kidney transplantation. Finally, 82 patients were included in the analysis.

The patients included in the analysis were predominantly male, of mixed race, with a mean age of 48.8 ± 14.9 years, and only 12.2% were older adults. Hypertension was prevalent comorbidity (82.9%), and two-thirds of the patients exhibited polypharmacy. Most patients received grafts from deceased donors (Table 1).

**Table 1:** Clinical and demographic characteristics of kidney transplant candidates

Variable	n	Total n = 82	Non-Frail n = 30	Frail n = 52	P value
**Age (yo),** mean ± SD	82	48.8±14.9	46.2±14.0	50.3±15.3	0.130
**Older adult (> 65 yo),** n(%)	82	10 (12.2%)	3 (10.0%)	7 (13.5%)	0.739
**Male gender,** n(%)	82	66 (80.5%)	26 (86.7%)	40 (76.9%)	0.284
**Race,** n(%)	82				0.090
Caucasian		11 (13.4%)	4 (13.3%)	7 (13.5%)	
Mixed race		63 (76.8%)	20 (66.7%)	43 (82.7%)	
Afro-Brazilian		7 (8.5%)	5 (16.7%)	2 (3.8%)	
Asian		1 (1.2%)	1 (3.3%)	0 (0.0%)	
**Educational level,** n(%)	80				>0.968
College		16 (20.0%)	6 (20.0%)	10 (20.0%)	
High School		32 (40.0%)	13 (43.3%)	19 (38.0%)	
Elementary School		27 (33.8%)	9 (30.0%)	18 (36.0%)	
Illiterate		5 (6.3%)	2 (6.7%)	3 (6.0%)	
**Time on dialysis (months),** median (IQR)	82	38.0 (24.0-54.0)	38.5 (25.0-58.5)	38.0 (22.8-51.8)	0.447
**CKD etiology,** n(%)	82				0.102
Diabetes Mellitus		20 (24.4%)	3 (10.0%)	17 (32.7%)	
Hypertension		18 (22.0%)	6 (20.0%)	12 (23.1%)	
Glomerulopathy		15 (18.3%)	5 (16.7%)	10 (19.2%)	
PKD		8 (9.8%)	5 (16.7%)	3 (5.8%)	
Other		7 (8.5%)	4 (13.3%)	3 (5.8%)	
Unknown		14 (17.1%)	7 (23.3%)	7 (13.5%)	
**Comorbidities**					
CHF, n(%)	82	12 (14.6%)	4 (13.3%)	8 (15.4%)	>0.999
Hypertension, n(%)	82	68 (82.9%)	21 (70.0%)	47 (90.4%)	**0.018**
Diabetes mellitus, n(%)	82	28 (34.1%)	7 (23.3%)	21 (40.4%)	0.117
Depression, n(%)	82	3 (3.7%)	0 (0.0%)	3 (5.8%)	0.295
Pneumopathy, n(%)	82	2 (2.4%)	0 (0.0%)	2 (3.8%)	0.530
Rheumatic disease, n(%)	82	8 (9.8%)	0 (0.0%)	8 (15.4%)	**0.024**
Polypharmacy*, n(%)	72	46 (63.9%)	16 (53.3%)	30 (71.4%)	0.115
**BMI (Kg/m2),** mean ± SD	82	24.9±4.3	25.3±4.6	24.6±4.2	0.427
**Deceased donor KT,** n(%)	82	73 (89.0%)	26 (86.7%)	47 (90.4%)	0.718

BMI = body mass index; SD = standard deviation; IQR = interquartile range; CKD = chronic kidney disease; KT = kidney transplant; PKD = polycystic kidney disease; CHF = congestive heart failure. *Current use of five or more drug classes.

### Frailty phenotype

**Figure 1:**
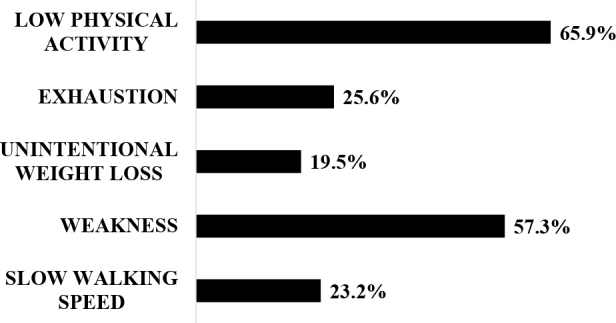
Performance of kidney transplant candidates in the five parameters assessed for frailty diagnosis.

Figure 1 illustrates the performance of KT candidates in the five components assessed using the instrument for diagnosing physical frailty. The most affected domains were physical activity (65.9%), weakness (57.3%), and self-reported exhaustion (25.6%). Thirty patients were considered non-frail (Non-frail group, 36.6%), 28 (34.1%) intermediate, and 24 (29.3%) frail (Frail group, n = 52, 63.4%).

### Factors associated with frailty phenotype

Frail patients had a higher prevalence of hypertension (90% vs. 70%, P = 0.018) and rheumatic diseases (15% vs. 0%, P = 0.024) (Table 1). No other significant differences were observed between the groups in terms of age, sex, race, educational level, body mass index, kidney disease etiology, or dialysis duration.

**Figure 2:**
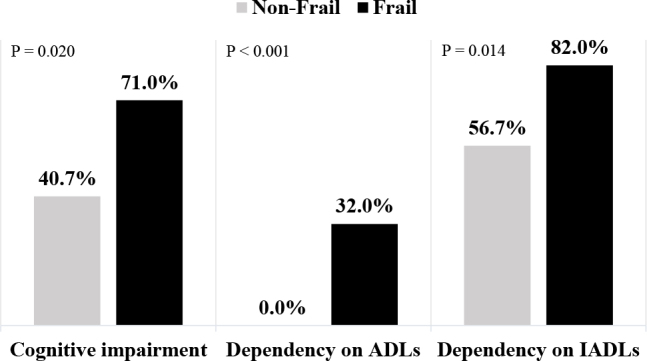
Conditions associated with frailty phenotype.

**Figure 3:**
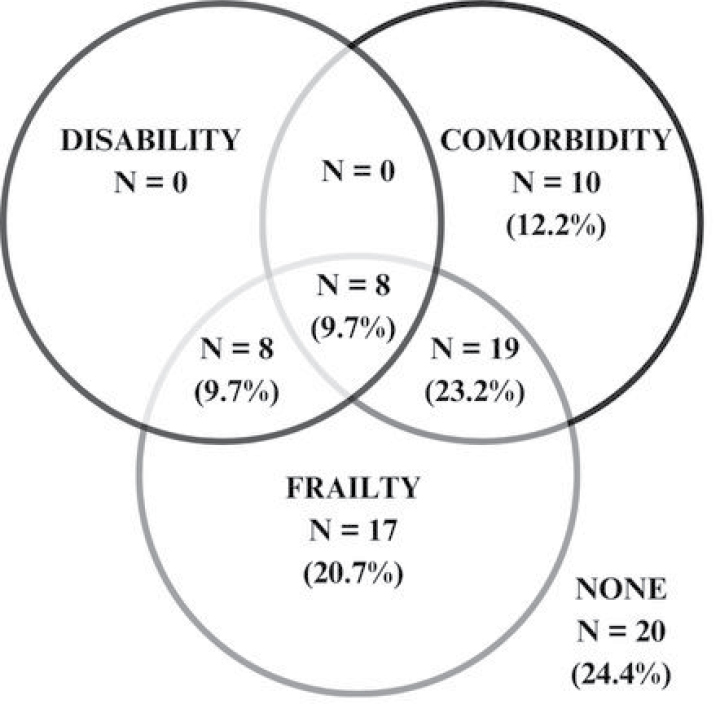
Venn diagram showing the overlap between frailty, comorbidities, and functional disability.

Cognitive impairment was more frequent in frail/intermediate patients than in non-frail patients (71.0% vs. 40.7%, P = 0.020). This group also showed greater dependence on basic (32% vs. 0%, P < 0.001) and instrumental (82.0% vs. 56.7%, P = 0.014) activities of daily living (Figure 2). As demonstrated in the Venn diagram, an overlap was observed between frailty, comorbidities, and disability (Figure 3).

ADLs = activities of daily living; IADLs = instrumental activities of daily living

58 patients were assessed for cognitive impairment and 80 patients for dependence on ADLs and IADL

Frailty: patients with a Fried score ≥ 2; Comorbidity: patients with two or more comorbidities beyond chronic kidney disease; Disability: patients with a Katz score ≥ 1.

As for laboratory tests, frail patients had lower mean hemoglobin (11.9 ± 2.7 vs. 13.4 ± 1.8 g/dL, P = 0.005) and median serum creatinine levels (7.0 vs. 9.0 mg/dL, P = 0.044) (Table 2).

**Table 2:** Laboratory tests collected in the pre- kidney transplant evaluation routine

Laboratory tests	n	Non-Frail n = 30	Frail n = 52	P value
**Hemoglobin** (g/dL), mean ± SD	82	13.4 ± 1.8	11.9 ± 2.7	**0.005**
**Leucocytes** (cells/mm^3^), mean ± SD	82	7,645 ± 2,556	6,662 ± 3,206	0.114
**Platelets** (cells/mm^3^), median (IQR)	82	171,600 (139,350 – 232,900)	158,250 (117,125 – 200,600)	0.101
**Creatinine** (mg/dL), median (IQR)	82	9.1 (7.0 – 11.0)	7.1 (6.0 – 10.0)	**0.044**
**Albumin** (g/dL), median (IQR)	76	4.0 (3.0 – 4.0)	3.9 (3.0 – 4.1)	0.316
**Calcium** (mg/dL), median (IQR)	73	9.0 (8.7 – 10.1)	9.1 (8.6 – 9.7)	0.742
**Phosphorus** (ng/dL), median (IQR)	73	5.3 (3.5 – 6.1)	4.9 (3.6 – 5.8)	0.336
**PTH** (ng/dL), median (IQR)	67	165 (8.0 – 679.3)	134 (22.8 – 375.0)	0.685
**Vitamin D** (U.I), median (IQR)	45	224 (44.0 – 386.0)	211 (39.6 – 368.8)	0.666
**Cholesterol** (mg/dL), median (IQR)	71	150 (114.5 – 182.3)	148 (117.0 – 176.0)	0.825
**Triglycerides** (mg/dL), median (IQR)	75	77 (40.0 – 199.0)	78.5 (35.3 – 146.3)	0.594
**Iron** (mmol/mL), median (IQR)	55	117 (62.0 – 171.0)	128.5 (61.8 – 195.3)	0.795
**Ferritin** (ng/mL), median (IQR)	50	48 (28.5 – 586.5)	190 (41.0 – 457.5)	0.454
**CRP** (mg/L), median (IQR)	42	8 (2.0 – 27.0)	20 (4.1 – 50.0)	0.300

KT = kidney transplant; PTH = parathyroid hormone; CRP = C-reactive protein; SD = standard deviation; IQR = interquartile range–

## DISCUSSION

This study revealed a high prevalence of frailty among patients with ESKD who were listed and deemed eligible for a KT. Additionally, the frailty phenotype was associated with dependence on both basic and instrumental activities of daily living, cognitive impairment, anemia, and lower creatinine levels, which are potentially indicative of sarcopenia. We highlight that frailty was evaluated immediately before KT surgery despite the personnel and time burdens. Furthermore, our samples shared key epidemiological characteristics (age, sex, CKD etiology, and deceased donors) with the global population of KT recipients.

Nearly one-third of the studied patients (29.3%) were frail at the time of the KT surgery, reaching 63.4% when intermediately frail individuals were included. These prevalence rates are higher than those reported both globally (11–20%) and in Brazil (16%).^12^ The prevalence of frailty depends on the selected diagnostic tool and, consequently, the assessed domains.^22^ Furthermore, the prevalence within our study population was higher than those of other studies utilizing the same diagnostic tool.^1,12^ Compared with Brazilian data from Dos Santos Mantovani et al.,^11^ our sample population was older and had a higher percentage of patients with diabetes, which may justify this finding. However, unmeasured variables, such as social conditions and nutritional status, may have influenced this result.

As previously reported, the main components of the frailty phenotype were physical inactivity and weakness.^17^ Notably, 65% of patients were inactive, demonstrating the potential for intervention in this modifiable domain, including implementing physical activities during the dialysis session.^22^

Arterial hypertension and rheumatological diseases were associated with frailty. The link between frailty and comorbidities is well-known.^12^ However, studies have significant heterogeneity with regard to the association between each comorbidity alone and the frailty phenotype in waitlisted patients with ESKD.^12^ Detection of the extent to which frailty is a cause or consequence of some comorbidities is also challenging.^22,23^

In patients with CKD, cognitive function declines as glomerular filtration rate decreases. Cognitive impairment has been described in up to 51% of patients undergoing dialysis,^24^ and is a risk factor for a lower likelihood of being listed for a KT, longer time to transplant listing, lower transplant rates, and death.^25^ In our study, frail patients were more likely to present with cognitive impairment, as previously reported.^26^ Subclinical cognitive impairment evaluation is time-consuming and not included as a routine for listing or eligibility decisions for transplantation. Frail patients in our cohort also exhibited a greater extent of morbidity and dependence on basic and instrumental activities of daily living, as consistently demonstrated in previous studies.^26^

Frail patients had reduced hemoglobin levels, which has been previously described and are likely related to malnutrition and inflammation.^27,28^ Additionally, these patients displayed lower creatinine values, possibly indicative of sarcopenia, given that they were all adults and were demographically similar in terms of age, sex, and body mass index. We found no association between frailty and inflammation, iron deficiency, or mineral or bone disorders. The sample size may have constrained this analysis.^29^

This study has some limitations. Although two transplant centers were included, both located in public hospitals within the same Brazilian city, the potential for demographic homogenization exists, restricting the extrapolation of findings to populations with diverse characteristics. Despite being adequate to estimate the prevalence of frailty,^11^ the sample size limits inferential analyses, which are exploratory. However, a significant challenge in evaluating frailty and associated conditions before transplantation is their dynamic nature, which requires assessment immediately before transplantation, as performed in this study. In institutions that primarily conduct deceased donor transplants, this poses a challenge because of the logistical complexity of promptly operationalizing the necessary tests without causing delays in the transplant process.

Despite these challenges and limitations, this study is pioneering in evaluating the prevalence and factors associated with frailty among patients with ESKD. The study provides critical guidance for addressing this population from a public health perspective, including the development of frailty prevention programs that incorporate early detection through cognitive and functional screening along with nutritional and exercise interventions to enhance physical and mental health. Additionally, implementing immediate pretransplant frailty assessments may rapidly identify at-risk patients, allowing for tailored perioperative management and potential reduction in postoperative complications such as delayed graft function, acute rejection, and graft loss. These findings are highly relevant to transplant services, offering a deeper understanding of the issue and suggesting actionable strategies for diagnosing and addressing frailty during pretransplant evaluations.

## CONCLUSION

We found a high prevalence of frailty among ESKD patients listed and eligible for KT. Furthermore, the frailty phenotype was associated with physical disability, cognitive impairment, and laboratory findings related to sarcopenia. These findings highlight the need to include early frailty assessments and rehabilitation measures before KT to prevent adverse outcomes.
